# Fracture resistance of pressed ZLS crowns versus pressed LD crowns under thermo-mechanical cycling

**DOI:** 10.1590/0103-6440202204993

**Published:** 2022-12-05

**Authors:** Basma Osama Salem, Dina Magdy Elshehawi, Gihan Abdelhady Elnaggar

**Affiliations:** 1 Faculty of dentistry, Cairo university, department of fixed prosthodontics, Giza, Egypt.

**Keywords:** cyclic loading, fracture resistance, lithium disilicate

## Abstract

The aim of this study was conducted to assess the fracture resistance of zirconia reinforced lithium silicate all ceramic material “Celtra Press” compared to lithium disilicate one “IPS e-max Press” under simulated oral conditions. Fourteen ceramic crowns were fabricated on epoxy dies which were duplicated from stainless steel master die and divided into two equal groups (n=7) according to the material of construction; Group A: Crowns fabricated with IPS e-max Press material and Group B: Crowns fabricated with Celtra Press material. The crowns were then cemented onto their corresponding dies with a self-adhesive resin cement and subjected to thermocycling and cyclic loading. Then they were loaded to fractur in a universal testing machine. The results were tabulated and statistically analyzed using Kolmogorov-Smirnov and Shapiro-Wilk tests. Student t-test used to compare mean values. The significance level was set at P ≤ 0.05 and 95% Confidence interval. Statistical analysis was performed using Graph Pad Instat (Graph Pad, Inc.) software for windows. The mean ± SD values of fracture resistance were recorded for lithium Disilicate group (1706.01 ±154.32 N) meanwhile the mean ± SD value recorded with celtra group were (1550.67±196.71 N). Zirconia reinforced lithium silicate ceramic crowns produced comparable fracture resistance values to lithium disilicate ceramic crowns and both tested materials are within the clinically acceptable values in the posterior area.

## Resumo

Este estudo teve como objetivo avaliar a resistência à fractura do silicato de lítio reforçado com zircônio todo o material cerâmico "Celtra Press" em comparação com um "IPS e-max Press" em condições orais simuladas. Quatorze coroas cerâmicas foram fabricadas em moldes epóxi que foram duplicados a partir de moldes principais de aço inoxidável e divididos em dois grupos iguais (n=7) de acordo com o material de construção; Grupo A: Coroas fabricadas com material IPS e-max Press e Grupo B: Coroas fabricadas com material Celtra Press. As coroas foram então cimentadas nos seus moldes correspondentes com um cimento de resina auto-adesivo e submetidas a ciclos térmicos e cargas cíclicas. Em seguida, foram carregadas para fraturar numa máquina universal de ensaios. Os resultados foram tabulados e analisados estatisticamente utilizando os testes Kolmogorov-Smirnov e Shapiro-Wilk. O teste t de Student usado para comparar os valores médios. O nível de significância foi fixado em P ≤ 0,05 e intervalo de confiança de 95%. A análise estatística foi realizada utilizando o software Graph Pad Instat (Graph Pad, Inc.) para Windows. Os valores médios ± SD da resistência à fractura foram registados para o grupo Disilicate de lítio (1706,01 ±154,32 N) enquanto que os valores médios ± SD registados com o grupo celtra foram (1550,67±196,71 N). As coroas cerâmicas de silicato de lítio reforçado com zircónio produziram valores de resistência à fractura comparáveis aos das coroas cerâmicas de dissilicato de lítio e ambos os materiais testados estão dentro dos valores clinicamente aceitáveis na área posterior.

## Introduction

All ceramic restorations have been considered as an important treatment option in fixed prosthodontics compared to metal ceramic restorations. This could be attributed to their increased esthetics properties and recorded strength. Therefore, all ceramic materials have been widely used to fabricate the restorations used in anterior and posterior areas.

Recent advances in ceramic processing methods have been made to simplify the work of the dental technician and allow for greater quality control for ceramic materials, which has increased their mechanical reliability. Among those advances pressable ceramics that have been introduced and widely used due to the ease of fabrication and high esthetics of the final restoration in addition to better marginal adaptation but still trials are made to enhance the mechanical properties without compromise the esthetics of the final restorations.

The pressable ceramic materials were suggested to have higher mechanical properties when compared to the CAD-CAM fabricated ones. The literatures claimed the superior mechanical performance of the pressing technique of construction to the avoidance of the hard milling which occurs in the CAD-CAM systems and induces a cascade of events on the ceramic surface and subsurface resulting in; rough surfaces, radial and lateral cracks, chipping, damage, and residual stress introduction. All of these factors constitute potential sites for fracture initiation and consequent failure of the CAD-CAM restorations compared to the pressed one (1-3). Lithium disilicate "IPS e-max Press" was introduced to the market at 2005 to enhance the strength of the pressable ceramics by its crystalline lithium disilicate structure. The microstructure of IPS e-max Press consists of approximately 70% lithium disilicate crystals measuring 3 to 6 mm in length. However, the strength of this material is still compromised to be used in high stress bearing areas (4).

Zirconia reinforced lithium silicate- ZLS "Celtra Press (Sirona Dentsply, Milford, DE, USA)" is the new generation of high strength glass ceramics. The introduction of zirconia in each microstructure was said by its manufacture to enhance its strength to give exceptional flexural strength more than 500 MPa. It contains a homogeneous crystalline structure made of lithium silicate crystals, and reinforced with tetragonal zirconia fillers (about10% by weight) (5).

However, there is no enough investigation on this zirconia reinforced lithium silicate pressable ceramic material to make its use more reliable in high stress bearing areas. That is why it was used in this investigation as this material should be accurately tested to approve the claims of the manufacture regarding its high fracture strength, as this will affect clinician's choice in restoring teeth in the high stress bearing areas such as restoring posterior teeth with high masticatory loads (6).

Therefore, the present study was conducted to assess the fracture resistance of zirconia reinforced lithium silicate pressable ceramic material compared to the lithium disilicate pressable ceramic material (control group) under simulated oral conditions as it was suggested in the literature (6,7) that further investigations are needed to evaluate this ZLS ceramic material.

The hypothesis tested in the present study was that the fracture resistance of zirconia reinforced lithium silicate "Celtra Press" all ceramic crowns would be higher than that of lithium disilicate "IPS e-max Press" crowns under simulated oral conditions (thermal cycling and cyclic loading).

## Materials and methods

Sample size calculation was performed using G*Power Version 3.1.9.2. Fourteen specimens simulating the prepared maxillary upper first premolar was calculated according to the power analysis, which was calculated by using the fracture resistance as the primary outcome. The effect size d = (1.843) was calculated based upon the results of Hamza, T. A., & Sherif, R. M (2019) (8). Using alpha ($\alpha$) level of 5% and Beta ($\beta$) level of 20% "i.e., power = 80%"; the minimum estimated sample size was a total of 12 specimens. The sample size was increased to a total of 14 specimens (7 specimens per group) to compensate for the use of non-parametric tests.

A total of 14 ceramic restorations were fabricated; they were divided into two equal groups (n=7), each according to the type of ceramic material used. Group A; monolithic lithium disilicate (IPS e.max Press) and group B; monolithic zirconia-reinforced lithium silicate ceramics (Celtra Press).

Stainless steel die was constructed with an engineering lathe (Automatic feedback lathe-BV20B-L; Jaixing Datong Machinery Co. Ltd.,China) to represent a prepared upper first premolar teeth receiving all ceramic crowns. The stainless-steel die was constructed with 10–12 degree taper, 4 mm height and a 1-mm-wide deep chamfer finish line. The machined stainless-steel die was made with V-shape occlusal surface to ensure accurate repositioning of the crowns, prevent their rotational movement and simulate the buccal and palatal cusps of the natural dentition (8).

Stainless steel die was duplicated with silicone impression material then poured in epoxy resin material (Kemapoxy; CMB Intl. Giza, Egypt) which was mixed according to the manufacturer's recommendation, and poured into a silicone mold (Replisil; Zubler USA, Dallas, TX) under vibration. Fourteen epoxy dies were made and left to polymerize in place for 24 hours.

The prepared model was scanned using 3D extraoral Scanner (Medit Identica T500 Dental) after spraying the stainless-steel die with a reflective powder (3D Renfert-Scanspray Powder; RENFERT Dentaltix company) to enhance the precision of the optical impressions acquired by creating a uniformly reflective surface then STL files were produced using the appropriate software.

A 3D model was created, after evaluating the clarity of the scan then the margins were identified, and the path of insertion was determined to prepare the restoration for editing. The cement space was set to 50 µm according to the manufacture recommendation and the buccolingual, the mesiodistal dimensions, and the cusp heights of the restoration outline were drawn on the design window then CAD-CAM milled wax pattern was fabricated using CAD-CAM wax blanks (Bilkam CAD-CAM wax blanks) and a five axis milling machine (K5+ five axis milling mchine,VHF camfacture AG, Germany) (9,10).

A total of 14 ceramic restorations were fabricated; they were divided into two equal groups (n=7), each according to the type of ceramic material used. Group A; monolithic lithium disilicate (IPS e.max Press) and group B; monolithic zirconia-reinforced lithium silicate ceramics (Celtra Press).

Crowns were pressed using the milled wax pattern. Spruing, investing and divesting were made according to manufacture instructions of each material. The investment of the lithium disilicate crowns was carried out with IPS e-max phosphate bonded investment material (IPS® PressVest Premium, Ivoclar Vivadent; Obsidian, Glidewell Laboratories). While for zirconia reinforced lithium silicate crowns was carried out with Celta press phosphate bonded investment material (Celtra Press investment, Dentsply Sirona; DeguDent GmbH, Germany).

Power firing of Celtra press crowns was made to obtain the maximum strength as recommended by the manufacture after complete divesting of the pressed crowns then glazing of the ceramic crowns of both groups was made according to the manufacture instructions of each material.

The fitting surface of each crown was etched with 9.5% hydrofluoric acid gel (Porcelain etchant, Bisco. inc. schumburg. U.S.A) for 20 second then thoroughly rinsed with water, and air-dried. The etched surface was coated with a silane coupling agent (Porcelain primer, Bisco. inc. schumburg.U.S.A) which was applied with a brush and air thinned after 1 minute. After that, crowns were cemented using self-adhesive dual cure resin cement (Calibra universal dentsply, Caulk, Milford, DE, USA) (11,12).

The activation, mixing, placement, and polymerization followed the manufacturer's recommendations. Mixing of the cement was done using automatic mixing tip to ensure the consistency of the cement. Each restoration was seated on its corresponding epoxy resin die and fixed to a specially designed cementation device for load application during the cementation procedure. Crowns were maintained under constant static load of 5 kg for 5 minutes using the load applicator device then all specimens were exposed to tack light curing for 2 seconds and the excess cement was removed with a scaler. After that light curing was completed for 20 second for each side until complete curing of the cement then all specimens were stored in normal saline at room temperature for 24 hours until testing (8,13).

The ceramic crowns of both tested groups were then subjected to 2500 thermal cycles. This number of thermal cycles was equivalent to three months of the clinical service. (14) The thermo cycling was made with Robote thermocycling machine (Robota automated thermal cycle; Bilge, Turkey). Each cycle included the immersion of the samples in high temperature and low temperature water baths. The dwell times were 25 s. in each water bath with a lag time 10 s. in between the two water baths. The low-temperature point was 5°C. The high temperature point was 55°C. which was simulating the clinical situation (14-16)

After that, the mechanical aging of the specimens was performed using a programmable controlled equipment (Robota chewing simulator, Model Ach-09075dc-T, Ad-Tech Technology Co., Ltd., Germany). ROBOTA chewing simulator has four chambers simulating the vertical and horizontal movements simultaneously in the thermodynamic condition. Specimens were subjected to a weight of 5 kg with 3 mm vertical movement, 1mm horizontal movement at 1.6 Hz frequency and 2.4 N.m torque which was comparable to 49 N of chewing force. The test was repeated 37500 times to clinically simulate the 3 months chewing condition, according to previous studies (15,17).

All samples were individually mounted on a computer-controlled material testing machine (Model 3345; Instron Industrial Products, Norwood, MA, USA) with a loadcell of 5 kN and data were recorded using a computer software (Instron® Bluehill Lite Software). Samples were secured to the lower fixed compartment of testing machine by tightening screws. Fracture test was done by compressive mode of load which was applied occlusally using a metallic rod with round tip (3.8 mm diameter). The metallic rod was attached to the upper movable compartment of the testing machine which was traveling at a cross-head speed of 1 mm/min.

A tin foil sheet was placed between the load applicator and the specimen to achieve homogenous stress distribution and minimize the transmission of the local force peaks. The load at failure manifested by an audible crack and confirmed by a sharp drop at the load-deflection curve, which was recorded on the computer software. The load required to fracture was recorded in Newton (8,13).

Statistical analysis was performed using Graph Pad Instat (Graph Pad, Inc.) software for windows. Data were presented as mean, standard deviation (SD), range (Minimum – Maximum) for values. Data were explored for normality by checking the data distribution and using Kolmogorov-Smirnov and Shapiro-Wilk tests. Student t-test used to compare mean values. The significance level was set at $P \leq 0.05$ and 95% Confidence interval.

## Results

The lithium disilicate "IPS e-max press" ceramic group recorded statistically non-significant higher fracture resistance mean value than the zirconia reinforced lithium silicate "celtra press" ceramic group as indicated by t-test.

Descriptive statistics showing mean values and standard deviation of fracture resistance test results measured in Newton (N) as function of material groups were summarized in Table 1 and graphically drawn in Figure 1.

**Table 1.** Results (Mean±SD) of the effect of the ceramic type on the fracture resistance of both tested groups.

| Variables | | Mean | SD | 95% CI | | Range | |
|---|---|---|---|---|---|---|---|
| | | | | Lower | Upper | Min. | Max. |
| Material group | E.max ceramic group | 1706.01 | 154.32 | 1544 | 1868 | 1525 | 1953.2 |
| | Celtra press ceramic group | 1550.67 | 196.71 | 1344.2 | 1757.1 | 1232 | 1745 |
| t-test | t-value | | | 1.5 | | | |
| | P value | | | 0.1590 NS | | | |

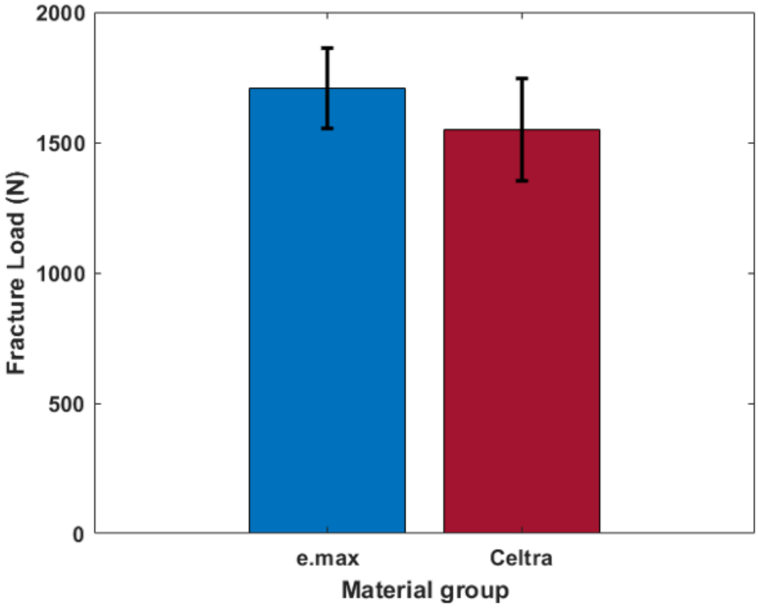

**Figure 1.** Bar chart showing the mean values of fracture resistance for both ceramic groups

## Discussion

In the present investigation, IPS e.max press was used as the control group. It is a lithium-disilicate pressable ceramic material which combines the advantages of esthetic and high fracture resistance. That's why, this material is widely used in clinical practice.

However, the utilization of lithium disilicate in high stress bearing areas is a controversial issue. The literature data is highly variable in reporting its survival and success rates, ranging from rather poor clinical results to acceptable long-term serviceability both in anterior and posterior sites (18).

The hypothesis that the fracture resistance of zirconia reinforced lithium silicate all ceramic "Celtra Press" crowns will be higher than that of lithium disilicate "IPS e-max Press" crowns under simulated oral conditions (thermal cycling and cyclic loading) was rejected. It was found that lithium disilicate "IPS e.max press" ceramic group recorded statistically non-significant higher fracture resistance mean value than zirconia reinforced lithium silicate "Celtra Press" cermic group.

The non-significant difference in the fracture strength between the two different ceramic groups could be related to the low thermal expansion which occurs during the manufacture processing of the IPS e-max press compared to the Celtra press as Celtra press material needs an extra firing cycle compared to IPS e-max press to obtain its final strength 'what is known with power firing cycle'. Therefore, the repeated firing cycles of the celtra press material could affect the final fracture load values (19).

Also, despite of the manufactures claims that adding zirconia fillers particles to Celtra press material will reinforce the ceramic structure by crack interruption; There is another important factor that greatly affect the strength of the ceramic material which is the number of crystals filler in the material. Celtra Press material has only 10% by weight of dissolved zirconia reinforcing the glass matrix. According to manufacturer information, crystallized Celtra Press has lower crystals filler volume (36% volume of lithium disilicate and lithium silicate) when compared to that of crystallized IPS e.max press (70% volume of lithium disilicate) (19,20).

In addition to, the presence of zirconia in the Celtra Press material decreases the amount of the glass matrix, which dissolves during etching. This could decrease the bond ability of the restoration and increase the grain boundaries that increase the crack initiation and propagation and subsequently decrease the fracture resistance compared to IPS e-max press material, which has a densely packed lithium disilicate crystalline structure which hinders the crack propagation (21).

Therefore, the non-significant difference between the two tested groups could be attributed to the decreased number of the crystal fillers in ZLS compared to LD, the decreased bond ability of ZLS due to the decreased amount of glass matrix available for bonding compared to LD and the increased number of firing cycles of ZLS crowns compared to LD crowns.

The findings of the present study were in agreement with Al-akhali et al. (2017), Schwindling et al. (2017), Taha, D. et al. (2018) and Furtado de Mendonca et al (2019) who reported non-significant difference in the fracture resistance between the lithium disilicate and the zirconia reinforced lithium silicate materials (18,13,22).

However, the result of the present study was opposed by Hamza, T. A., & Sherif, R. M (2019) who reported a significant higher fracture resistance of zirconia reinforced lithium silicate compared to lithium disilicate but this could be related to the absence of thermal cycling and the decreased number of cyclic loading cycles "only 10.000 cycles" which could affect the final results of the fracture resistance test (8).

The result of the present study was also opposed by Ghajghoui & Tasar-Faruk (2019) who reported a significant higher fracture resistance of zirconia reinforced lithium silicate compared to lithium disilicate but this could be related to the absence of cyclic loading as the samples in his investigation were only subjected to thermal cyclic which is not simulating the clinical situation and may affect the final results of the fracture resistance test (23).

Also, Zarone et al. (2019); Kashkari et al. (2019) reported a significant higher fracture resistance of lithium disilicate compared to zirconia reinforced lithium silicate rather than the non-significant difference which was reported in the present study, and this could be related to the reduced sample size in his investigation "only 3 samples in the group of ZLS" and also because he did not follow the manufacture recommendation in the processing of ZLS material as he did not make the power firing cycle of the Celtra DUO material which could affect its final fracture strength (7,20). In the present study, the mean fracture loads for the tested groups were beyond the mean reported maximum masticatory forces which means that the ceramic crown materials used in the current study could withstand the maximum intraoral posterior masticatory forces. As the reported mean values of the maximum bite forces in the molar region were 847 N for men and 597 N for women and could reach 800 N for bruxer patients (18,24,25).

Finally, the present study is an invitro testing which gives an idea about the clinical expectation of the newly introduced dental materials before their use in the clinical practice; however clinical trials should be the final determinant to the performance of these materials.

## Conclusions

Within the limitations of this invitro study the following conclusions could be drawn:

1. Zirconia reinforced lithium silicate ceramic crowns produced comparable fracture resistance values to lithium disilicate ceramic crowns.
2. The fracture resistance of both tested materials (Zirconia reinforced lithium silicate and Lithium disilicate) is within the clinically acceptable values in the posterior area.
3. Both tested ceramic materials (Zirconia reinforced lithium silicate and Lithium disilicate) showed a favorable mode of fracture which enables their safe use in the oral cavity.

**Recommendations:**

1) Making a control group (storage in deionized water at 37o C for the period of mechanical cycling) for each type of ceramic and comparing with the results after the mechanical cycling test.

2) Further investigations should be carried out with more long-term simulation of the thermo-mechanical fatigue to simulate five years clinically and confirm the results of the present study.

3) Further investigations with failure analysis of both types of the ceramic crowns may be performed after the fracture resistance test.

4) Clinical investigations (invivo studies) should be conducted to assess the actual clinical success and the long-term durability of the newly introduced ZLS material "Celtre press ceramic material"

## Resumo

Este estudo teve como objetivo avaliar a resistência à fractura do silicato de lítio reforçado com zircônio todo o material cerâmico "Celtra Press" em comparação com um "IPS e-max Press" em condições orais simuladas. Quatorze coroas cerâmicas foram fabricadas em moldes epóxi que foram duplicados a partir de moldes principais de aço inoxidável e divididos em dois grupos iguais (n=7) de acordo com o material de construção; Grupo A: Coroas fabricadas com material IPS e-max Press e Grupo B: Coroas fabricadas com material Celtra Press. As coroas foram então cimentadas nos seus moldes correspondentes com um cimento de resina auto-adesivo e submetidas a ciclos térmicos e cargas cíclicas. Em seguida, foram carregadas para fraturar numa máquina universal de ensaios. Os resultados foram tabulados e analisados estatisticamente utilizando os testes Kolmogorov-Smirnov e Shapiro-Wilk. O teste t de Student usado para comparar os valores médios. O nível de significância foi fixado em $P \le 0{,}05$ e intervalo de confiança de 95%. A análise estatística foi realizada utilizando o software Graph Pad Instat (Graph Pad, Inc.) para Windows. Os valores médios ± SD da resistência à fractura foram registados para o grupo Disilicate de lítio (1706,01 ±154,32 N) enquanto que os valores médios ± SD registados com o grupo celtra foram (1550,67±196,71 N). As coroas cerâmicas de silicato de lítio reforçado com zircónio produziram valores de resistência à fractura comparáveis aos das coroas cerâmicas de dissilicato de lítio e ambos os materiais testados estão dentro dos valores clinicamente aceitáveis na área posterior.
